# Actions Taken by Bystanders During Sudden Cardiac Arrest: Analysis of Emergency Medical Service Documentation in Poland

**DOI:** 10.3390/jcm13247765

**Published:** 2024-12-19

**Authors:** Rafał Milewski, Jolanta Lewko, Gabriela Milewska, Anna Baranowska, Agnieszka Lankau, Magda Orzechowska, Elżbieta Krajewska-Kułak

**Affiliations:** 1 Department of Integrated Medical Care, Medical University of Bialystok, 15-096 Bialystok, Poland; anna.baranowska@umb.edu.pl (A.B.); agnieszka.lankau@umb.edu.pl (A.L.); magda.orzechowska@umb.edu.pl (M.O.); elzbieta.krajewska-kulak@umb.edu.pl (E.K.-K.); 2 Department of Primary Health Care, Medical University of Bialystok, 15-096 Bialystok, Poland; 3 Department of Reproduction and Gynecological Endocrinology, Medical University of Bialystok, 15-096 Bialystok, Poland; gabriela.milewska@umb.edu.pl

**Keywords:** out-of-hospital cardiac arrest, cardiopulmonary resuscitation, emergency medical team, intubation

## Abstract

**Background/Objectives**: Sudden cardiac arrest (SCA) is a severe medical condition involving the cessation of the heart’s mechanical activity. Following the chain of survival, which includes early recognition and calling for help, early initiation of cardiopulmonary resuscitation (CPR), early defibrillation, and post-resuscitation care, offers the greatest chances of saving a person who has experienced SCA. The aim of this study was to analyze cases of out-of-hospital cardiac arrest (OHCA) and assess the actions taken by bystanders. **Methods**: The input for analysis consisted of 49,649 dispatch records from the emergency medical team (EMT) at the Voivodeship Emergency Medical Station in Bialystok in 2018–2019. **Results**: Among the patients where bystanders performed CPR, the return of spontaneous circulation (ROSC) occurred in 30.53% of cases, whereas in the cases where the bystander did not perform CPR, ROSC occurred in 2.35% of cases. When cardiac arrest rhythm was ventricular fibrillation (VF) or pulseless ventricular tachycardia (pVT), ROSC occurred in 58.62% of cases, while there was asystole or pulseless electrical activity (PEA) present, ROSC occurred in 26.56% of cases. In patients who experienced OHCA in a VF/pVT rhythm and who underwent intubation, ROSC occurred in 58.73% of cases, whereas in patients who underwent alternative procedures for airway management, ROSC occurred in 83.33% of cases. **Conclusions**: The most significant factor influencing the occurrence of ROSC in patients is CPR initiation by bystanders. The presence of a rhythm that requires defibrillation increases the likelihood of achieving ROSC in the patient. Alternative methods for airway management appear to be more beneficial in VF/pVT rhythms.

## 1. Introduction

According to the World Health Organization, cardiovascular diseases constitute the most common cause of death as far as all of the recorded fatalities, both in Europe and worldwide, are concerned [1]. Among other factors, the rapid economic development and industrialization of most countries have contributed to an increase in the incidence of sudden cardiac arrest (SCA) [2]. SCA is a sudden and severe medical condition involving the cessation of the heart’s mechanical activity. It results in the stoppage of blood circulation, cessation of breathing, and consequently leads to irreversible brain damage [3]. In Poland, the incidence of out-of-hospital cardiac arrest (OHCA) is relatively high, amounting to 146 cases per 100,000 inhabitants annually [4]. Following the chain of survival, which includes early recognition and calling for help by bystanders, early initiation of cardiopulmonary resuscitation (CPR), early defibrillation, and post-resuscitation care, offers the greatest chances of saving a person who has experienced SCA [5]. There are four types of SCA rhythms: asystole and pulseless electrical activity (PEA)—both of which do not require defibrillation—and ventricular fibrillation (VF) and pulseless ventricular tachycardia (pVT)—where defibrillation is essential to restore a normal heart rhythm [6]. In the first minutes of cardiac arrest, VF occurs in 76% of cases [5]—the most favorable rhythm among rhythms in patients with SCA [7]. Untreated cardiac arrest leads to the patient’s death. In Poland, in cases of OHCA, resuscitation is performed by the emergency medical team (EMT) at the scene. The EMT do not initiate resuscitation if there are cardinal signs of death (postmortem lividity, postmortem coldness, postmortem rigidity, putrefaction, decomposition, skeletonization). When the return of spontaneous circulation (ROSC) is observed, the patient is transported to the hospital. ROSC was defined as the achievement of cardiac circulation with the presence of an organized heart rhythm with a pulse in the jugular artery at any point during the resuscitation attempt. Transporting a person with a SCA to hospital during resuscitation is permissible when there is a reversible cause of SCA. The EMTs perform medical emergency procedures (MEPs) recommended by the European Resuscitation Council (ERC). The EMTs use a manual defibrillator, secure the airway with a tracheal tube or supraglottic devices, ventilate using a manual resuscitator or respirator, use capnography, provide intravenous or intraosseous injection, and administer drugs such as adrenaline, amiodarone, lidocaine, oxygen, and apply fluid therapy [5].

The arrival of the EMT at the scene of an incident often takes too long. In most communities, the average response time for the EMT to reach the scene is 5 to 8 min [8,9]. A key factor in improving survival rates in the case of OHCA is the bystander’s actions within the framework of basic life support (BLS); it may triple survival rates along with favorable neurological outcomes [10,11]. Performing CPR ensures a small but significant circulation of blood to the brain and heart [12].

In the European countries, the survival rate for individuals who experience OHCA before reaching the hospital is very low, at just 8% [13].

The ERC emphasizes the ongoing development of the first aid provided by bystanders, including the use of mobile technologies and the implementation of social campaigns focused on training children, youth, and adults in CPR [14].

The aim of this study was to analyze cases of OHCA, assess the actions taken by bystanders in the event of cardiac arrest in the injured person, and analyze the procedures followed by the EMT in the cases of OHCA. Additionally, another aim was to evaluate the factors influencing ROSC in patients with OHCA.

## 2. Materials and Methods

### 2.1. Patients

**Figure 1:**
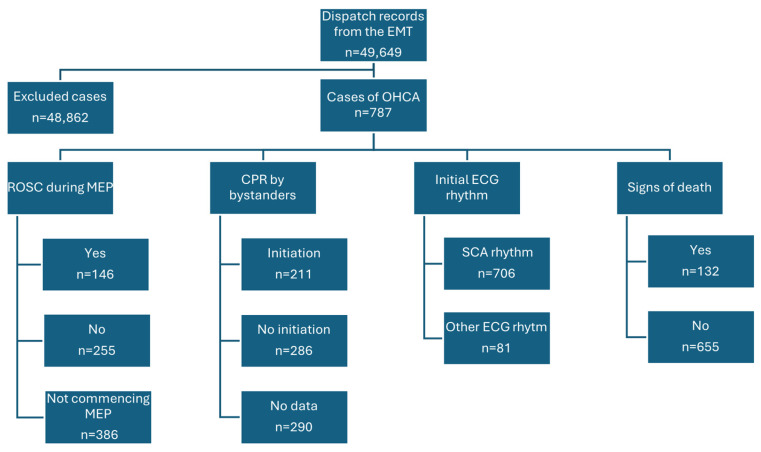
Flow chart with numbers of OHCA cases. The non-shockable SCA rhythm group included cases of asystole in patients with cardinal signs of death (n = 132). Other rhythms: sinus rhythm, supraventricular tachycardia (SVT), atrial fibrillation and atrial flatter (AF/AFl), and non-ST/ST-segment elevation myocardial infarction (NSTEMI/STEMI).

The input for analysis consisted of 49,649 dispatch records from the EMT at the Voivodeship Emergency Medical Station in Bialystok over a period of two years (2018–2019). This study covered the area of the City of Bialystok, which occupies an area of 102.13 km^2^ and has a population of approximately 298,000 inhabitants [15]. The inclusion criterion encompassed the cases in which a patient experienced cardiac arrest according to ICD-10 codes (I46, I49.0, R96, R98, R99) before EMT arrival or during EMT procedures. A total of 787 cases from the studied group met the above criteria. This group included cases of patients with cardinal signs of death (n = 132). The exclusion criteria were all ICD-10 codes other than those mentioned above or incorrectly completed dispatch records (Figure 1).

The data were collected according to a self-administered questionnaire completed by the researcher. The self-administered questionnaire consisted of 27 questions (Supplement S1), of which 17 questions came from the Utstein protocol. Data collected on the basis of the Utstein protocol were: time of call receipt, date, OHCA location, patient sex, patient age, bystanders, treatment before EMT arrival, chest compression, defibrillation with automated external defibrillator (AED), type of EMT, time that vehicle stopped, CPR initiation by the EMT, initial electrocardiogram (ECG) rhythm, shockable/non-shockable rhythm, defibrillation attempts, ROSC, and hospital admission. The analysis of the collected data was conducted, and the required assumptions were verified.

### 2.2. Statistical Analysis

In order to find correlations between qualitative characteristics, Pearson’s Chi-square test was used. The Mann–Whitney test was applied for comparing ordinal characteristics between two independent groups, while the Kruskal–Wallis test was used for more than two groups. Spearman’s rank correlation coefficients were also determined. The results were considered statistically significant at the *p* < 0.05 level. The data were collected in Microsoft Office Excel and subsequently subjected to statistical analysis using STATISTICA 13.3 by TIBCO Software Inc. (Krakow, Poland).

### 2.3. Bioethics Committee Approval

Approval for conducting this study was obtained from the Bioethics Committee of the Medical University of Bialystok, No R-I-002/610/2018, 20 December 2018.

## 3. Results

### 3.1. Distribution of OHCA

Based on the conducted research, it was found that the incidence of OHCA in the area of Bialystok was 133.1 per 100,000 residents per year.

**Figure 2:**
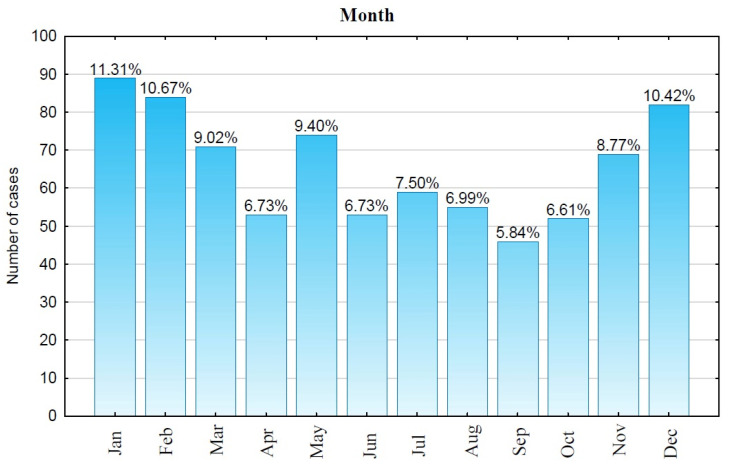
Distribution of OHCA cases on a monthly basis. Jan—January, Feb—February, Mar—March, Apr—April, Jun—June, Jul—July, Aug—August, Sep—September, Oct—October, Nov—November, Dec—December.

The highest number of cases was observed in the winter months: January (11.31%—89 cases), February (10.67%—84 cases), and December (10.42%—82 cases). This study indicated a lower number of cases in May (9.40%—74 cases), March (9.02%—71 cases), and November (8.77%—69 cases). Cardiac arrests were significantly less frequent during the summer months: July (7.50%—59 cases) and August (6.99%—55 cases). Even fewer OHCA cases occurred in April and June (6.73% each—53 cases), in October (6.61%—52 cases), and in September (5.84%—46 cases). The cases in which signs of death were observed were also taken into consideration (n = 787) (Figure 2).

**Figure 3:**
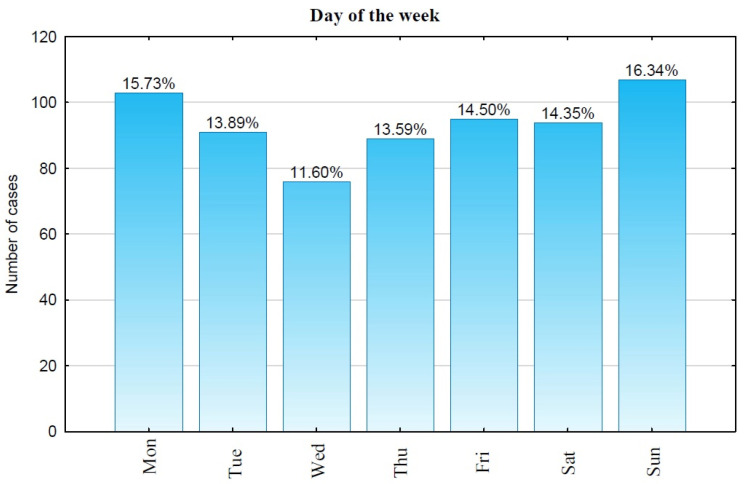
Distribution of OHCA cases by day of the week. Mon—Monday, Tue—Tuesday, Wed—Wednesday, Thu—Thursday, Fri—Friday, Sat—Saturday, Sun—Sunday.

OHCAs occurred least frequently on Wednesdays (11.60%—76 cases). Cases of OHCA were somewhat more common on Thursdays (13.59%—89 cases), Tuesdays (13.89%—91 cases), Saturdays (14.35%—94 cases), and Fridays (14.50%—95 cases). The highest number of OHCA cases in Bialystok occurred on Mondays (15.73%—103 cases) and Sundays (16.34%—107 cases). Only the cases without signs of death were taken into consideration (n = 655) (Figure 3).

#### 3.1.1. Distribution of OHCA Cases by Localization

OHCAs occurred most frequently in homes/apartments (78.27%—616 cases). Only about one in ten (9.78%—77 cases) patients who experienced cardiac arrest were in the street at that time. The remaining cases accounted for 11.94% (94 cases) of all of the locations where cardiac arrest occurred, which included the following areas: Social Welfare Home (12 cases), stairwell (9 cases), store (8 cases), clinic, construction site (6 cases each), hotel (5 cases), basement, sobering-up station, detention center, allotment gardens, park, beach (3 cases each), garage, workplace, sewage treatment plant, car workshop, homeless shelter, transport ambulance (2 cases each), shopping mall, train station, bus station, university (University of Bialystok), dormitory, school, government office, library, city stadium, wedding hall, fruit and vegetable market, convent, hospice, attic, hangar of a former military unit, forest, garbage shelter, and abandoned building/vacant property (1 case each). The cases in which signs of death were observed were also taken into consideration (n = 787).

#### 3.1.2. Distribution of OHCA Cases by Age and Sex

The numbers of OHCA cases in a variety of age and sex groups are presented in Table 1. OHCA occurred more frequently in men than in women (62.74%—490 cases). Just over one-third of the cases (37.26%—291 cases) occurred in women. In the case of six individuals, data on gender were not provided.

Cardiac arrests were most common among individuals aged 80–90 years (26.39%—205 cases). Cases were slightly less frequent in the 70–79 years age group (20.31%—157 cases) and the 60–69 years age group (19.79%—153 cases). A total of 103 individuals were aged 50–59 years, representing 13.32% of all cases. Fewer cardiac arrests were recorded in the following age groups: 40–49 years (7.37%—57 cases), over 90 years (6.34%—49 cases), 30–39 years (4.14%—32 cases), and 18–29 years (1.81%—14 cases). In 2018 and 2019, there were four cases of OHCA among children (individuals under 18 years of age). Based on the data, it is noteworthy that all of the individuals who experienced OHCA and were over 49 years old accounted for 86.16% of all the cases. In the case of 13 individuals, age was unknown, or their medical documentation lacked information; therefore, we excluded these cases from this analysis. The cases in which signs of death were observed were also taken into consideration. The average age for all the cases was 68.79 years, while the median age was 70 years (n = 774).

Considering the division into eight age groups (n = 770), it is evident that OHCA occurred proportionally more often in men than in women among the individuals under the age of 80. In contrast, in 80-year-old or older patients, women experienced cardiac arrest more frequently than men (Table 1).

The average age at which OHCA occurs was 65.01 years for men (median = 66 years, n = 481) and 75.03 years for women (median = 79 years, n = 289).

### 3.2. CPR Initiation by Bystanders

Approximately one in four bystanders (26.81%—211 cases) chose to perform CPR as part of BLS. In contrast, 36.34% (286 cases) of individuals did not initiate CPR for the person experiencing cardiac arrest. In 36.85% (290 cases) of incidents, the medical documentation did not provide information regarding that issue. The cases in which signs of death were observed were also taken into consideration (n = 787) (Table 2).

For the initiation of CPR as part of BLS, both chest compressions combined with rescue breaths and the performance of chest compressions alone were considered. Among the 211 cases of CPR performed by bystanders on individuals experiencing OHCA, as many as 80.57% (170 cases) included only chest compressions. In contrast, 19.43% (41 cases) opted for a two-component CPR approach, which included both chest compressions and rescue breaths.

An AED was used only four times before the EMT arrived. The device was operated by firefighters, detention center staff, and sobering-up station staff. Bystanders never used the AED.

Bystanders witnessing OHCAs rarely chose to perform CPR when signs of death were present in the patient (n = 132). Over half (55.30%—73 cases) correctly assessed the victim’s condition and refrained from initiating CPR. Despite the presence of signs of death, 15.91% (21 cases) of bystanders proceeded with CPR. In 28.79% (38 cases) of incidents, medical documentation did not provide information regarding that issue.

#### 3.2.1. The Relationship Between the Initiation of CPR by a Bystander and ROSC in the Patient

Our study demonstrated a statistically significant (*p* < 0.0001) relationship between the initiation of CPR by a bystander and the occurrence of ROSC and the patient’s transfer to the hospital. Among the patients where CPR was performed by a bystander, ROSC occurred in 30.53% (58/190) of cases, whereas in the cases where the bystander did not perform CPR, ROSC occurred in only 2.35% (5/213) of cases. Only the cases without signs of death were taken into consideration. In this analysis, we also excluded cases in which the medical records did not provide information regarding that issue (Table 2).

#### 3.2.2. The Relationship Between the Initiation of CPR by the Bystander and the Decision to Commence MEPs by the EMT

Our study revealed that bystander CPR initiation before EMT arrival increased the frequency of MEP initiation by the EMT (*p* < 0.0001). Among the patients who received CPR from a bystander, the EMT initiated MEPs in 67.89% (129/190) of cases. In contrast, among those where the bystander did not perform CPR, the EMT initiated MEPs much less frequently, in only 25.82% (55/213) of cases (in as many as 74.18% of cases, the EMT did not decide to start MEPs) (Table 2). Only the cases without signs of death were taken into consideration. In this analysis, we also excluded cases in which the medical records did not provide information about CPR initiation by bystanders.

#### 3.2.3. The Relationship Between the Patient’s Age and the Initiation of CPR by the Bystander

We observed that bystanders more frequently initiated CPR on younger patients. Among the adult patients aged 18–39 years, CPR was performed by bystanders in 58.82% of cases. For the patients in the age group of 40–64 years, CPR was initiated less frequently, in 53.06% of cases. Among the patients aged 65–80 years, bystanders performed CPR in 43.14% of cases, while for the patients over 80 years old, CPR was conducted before the arrival of the EMT in only 28.10% of cases (Table 2). The cases in which signs of death were observed were also taken into consideration. In this analysis, we also excluded cases in which the medical records did not provide information about CPR initiation by bystanders and patient’s age.

### 3.3. Time from the Acceptance of the Call by the Dispatcher to the Moment of Arrival of the EMT

Among all the cases involving dispatches of the EMT to patients experiencing OHCA, the time elapsed from when the dispatcher received the call to when the EMT arrived at the scene was 8–10 min (40.99%—291 cases). A slightly shorter time of 5–7 min was recorded less frequently, occurring in 33.24% (236 cases). A time frame of 11–13 min was observed 104 times, accounting for 14.65% of all cases. The remaining times fell into time groups, each constituting less than 7% of all cases (time groups: under 5 min—6.06%—43 cases; 14–16 min—3.24%—23 cases; over 16 min—1.83%—13 cases).

The average time was 8 min and 30 s, while the median was Me = 8 min (Q1 = 6 min, Q3 = 10 min). The cases in which signs of death were observed were also taken into consideration.

#### The Relationship Between the Time from the Acceptance of the Call by the Dispatcher to the Moment of Arrival of the EMT at the Scene and ROSC in the Patient

No statistically significant difference (*p* = 0.27) was found between the time from the acceptance of the call by the dispatcher to the arrival of the EMT at the scene and the occurrence of ROSC in the patient (along with the transfer to the hospital). The Mann–Whitney test was applied for comparing ordinal characteristics between two independent groups.

The average time from the acceptance of the call to the arrival of the EMT at the scene, in the cases where ROSC occurred, was 8 min and 27 s, while in the cases where ROSC did not occur, it was very similar at 8 min and 33 s. Only the cases without signs of death were taken into consideration.

### 3.4. ECG Rhythms in Cardiac Arrest

The most common initial ECG rhythm of SCA (n = 574) was asystole, observed in 74.56% (428 cases). Significantly less frequently, PEA was identified during the initial rhythm assessment in 15.33% (88 cases). Even less common was the presence of a rhythm requiring defibrillation—VF or pVT—which occurred in 10.10% (58 cases) (Table 3). Only the cases without signs of death were taken into consideration.

#### 3.4.1. The Relationship Between the Initial Rhythm of Cardiac Arrest and ROSC in Patients

Among the patients with SCA whose first observed rhythm of cardiac arrest was VF or pVT, ROSC occurred most frequently, at a rate of 58.62%. In the cases where the first rhythm was PEA, ROSC was significantly less common, occurring in only 31.43% of cases. The lowest occurrence of ROSC was observed in patients with asystole as the initial rhythm, with ROSC present in only 24.73% of cases. Only the cases without signs of death were taken into consideration. In this analysis, we also excluded cases in which the EMT did not initiate MEPs (Table 3).

Our results demonstrate that ROSC occurred more frequently when the first rhythm assessment conducted by the EMT revealed defibrillation rhythm. Among the patients whose first observed cardiac arrest rhythm was VF/pVT, ROSC occurred in 58.62% of cases, while among the other cardiac arrest rhythms (asystole and PEA), ROSC occurred in only 26.56% of cases. Only the cases without signs of death were taken into consideration. In this analysis, we also excluded cases in which the EMT did not initiate MEPs. In order to find correlations between qualitative characteristics, Pearson’s Chi-square test was used (*p* < 0.0001).

#### 3.4.2. The Relationship Between the Patient’s Age and the Initial Rhythm of Cardiac Arrest

**Figure 4:**
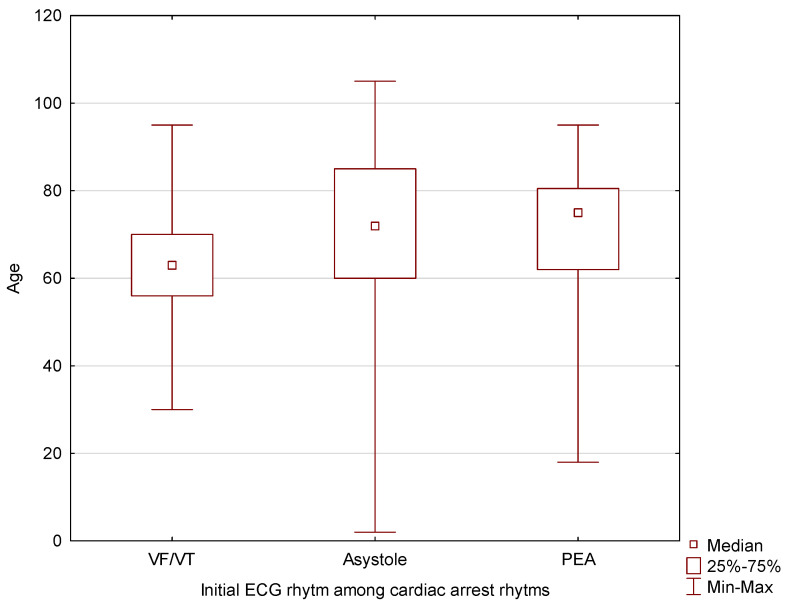
The relationship between the patient’s age and the initial ECG rhythm among cardiac arrest rhythms.

For the group where the first rhythm of cardiac arrest was VF/pVT, the median age was Me = 63 years (Q1 = 56 years, Q3 = 70 years). For the group where the first rhythm of cardiac arrest was asystole, the median age was significantly higher, at Me = 72 years (Q1 = 60 years, Q3 = 85 years). The highest median age was observed in the cases where the first rhythm of cardiac arrest was PEA, with a median of Me = 75 years (Q1 = 62 years, Q3 = 80.5 years). The obtained differences are presented in Figure 4. For statistical analyses, the Kruskal–Wallis test was used (*p* = 0.0002).

The conducted post-hoc analysis revealed statistically significant differences between the groups where the first rhythm of cardiac arrest was VF/pVT and asystole (*p* = 0.0001) as well as between the groups where the first rhythm of cardiac arrest was VF/pVT and PEA (*p* = 0.004). No statistically significant differences were found between the groups where the first rhythm was asystole and PEA. Only the cases without signs of death were taken into consideration.

#### 3.4.3. The Relationship Between Intubation and ROSC in Cases of Cardiac Arrest Where VF and/or pVT Was Present

Our study revealed a statistically significant relationship (*p* < 0.05) between the performance of tracheal intubation and the occurrence of the ROSC and transfer of the patient to hospital among the patients who experienced OHCA in a VF/pVT rhythm. Among the patients who underwent intubation, ROSC occurred in 58.73% of cases, whereas among the patients who had not undergone that procedure, ROSC was more frequent, occurring in 83.33% of cases. Only the cases without signs of death were taken into consideration.

#### 3.4.4. The Relationship Between Intubation and the Occurrence of ROSC in Cases of Cardiac Arrest Without the Presence of VF and/or pVT

No statistically significant difference (*p* = 0.83) was found between the performance of endotracheal intubation and the occurrence of ROSC and the transfer of the patient to hospital among the patients who experienced OHCA with rhythms other than VF/pVT (asystole and PEA). Among the patients who had undergone intubation, ROSC occurred in 28.57% of cases, while among the patients who had not undergone that procedure, ROSC occurred similarly in 29.79% of cases. Only the cases without signs of death were taken into consideration.

Among the cases of OHCA where there had been no signs of death and where the EMT had initiated MEPs for the patient, ROSC was achieved in 146 cases (36.41% of all cases) during the EMT’s resuscitation efforts, and the patient was transferred to hospital. In the remaining cases, where OHCA occurred (63.59%—255 cases), the EMT supervisor made the decision to discontinue advanced resuscitation efforts, which undoubtedly correlated with the occurrence of the patient’s death. This analysis also included cases in which a rhythm other than SCA was present during the initial ECG evaluation.

## 4. Discussion

In 2014, the incidence of all of OHCA cases in Europe was 84 per 100,000 inhabitants, while in Poland, that rate was 146 per 100,000 inhabitants [4]. Our analysis showed that the incidence of OHCA in Bialystok in the years 2018–2019 was 133.1 per 100,000 inhabitants annually. The highest number of OHCA cases was observed in the winter months, while the lowest number of cases was recorded in summer time [16]. In Bialystok, OHCA most frequently occurred on Sundays and Mondays, while they were least common on Wednesdays. In 2021, no significant changes in the number of OHCA cases based on the day of the week were observed in the United Kingdom [17]. In contrast, in the United States between 2005 and 2010, it was found that the highest number of OHCA cases occurred on weekends (i.e., Saturdays and Sundays) [18]. The most frequent localization of OHCA in Europe as well as in Bialystok and the Upper Silesia region is home [4,13,17,19]. Furthermore, the majority of OHCA cases in Poland, as well as in Europe, are men [4,13,20].

The average age for all OHCA cases in Bialystok in 2018–2019 was 68.79 years. Similarly, in Poland in 2018, it was 65.35 years [20]. In Europe, in 2014 and 2017, the average age for OHCA were as follows: 66.5 years and 67.6 years [4,13]. This investigation indicated that 86.16% of OHCA cases were in people over 49. As shown by the gender and age data, OHCA was more common in males under 80 than women. However, women experienced OHCA more frequently than males around 80 or older. The average age at which OHCA occurred for men was 65.01 years, while for women, it was 75.03 years. In comparison, 41% of all OHCA cases in the UK involve individuals between the ages of 15 and 64, while 57.6% involve patients over the age of 65. This is in contrast to Bialystok, where the average age of women who experience OHCS is 66.8 years and the average age of men who experience OHCA is 64.2 years [17].

We found that in the years 2018–2019 in Bialystok, on average, one in four individuals who witnessed an OHCA decided to perform CPR as part of BLS before the arrival of the EMT. In comparison, in 2018 in the Silesian Voivodeship, almost half of bystanders chose to initiate CPR [19,21]. In Europe, in 2014, 47.4% of bystanders to an event initiated CPR [4]. In 2017, that figure increased to 58% [13]. Both studies showed that in Europe, bystanders are increasingly choosing to perform CPR before the arrival of the EMT. The percentage share of bystanders performing CPR in Bialystok may be underestimated, as in 36.85% of the cases, the CPR records did not include the information about the actions taken by the bystander.

Our study found that bystanders were significantly more likely to perform CPR with chest compressions alone than with chest compressions and rescue breaths. These results were similar to those obtained in the Upper Silesia region [19]. Performing CPR with chest compressions along with breaths in Europe is undertaken slightly more often than in Poland [13]. Currently, it is recommended to perform only chest compressions on adults with cardiac arrest if the person providing first aid is not trained [14]; however, according to the EuReCa study, the combination of chest compressions and artificial ventilation results in statistically significant better outcomes for patient survival until hospital discharge (14% vs. 8%) [13].

In the cases where there were signs of death, more than half of the bystanders to OHCA correctly assessed the condition of the victim and did not initiate CPR. In 15.91% of cases, the bystanders conducted CPR despite the presence of signs of death. The decision to perform CPR by the bystanders, even in the presence of signs of death, was most likely due to a lack of medical knowledge, nervousness, stress, or the desire to save another person at all costs, even in the face of clear indications against BLS procedures. Nevertheless, it is important to commend those individuals who chose to attempt CPR, even in the cases involving the patients with signs of death.

Statistically significant higher rates of ROSC and hospital transfer were observed when a bystander initiated CPR. ROSC occurred in 30.53% of patients who had received CPR from a bystander, whereas it occurred in just 2.35% of patients who had not received CPR from a bystander. Our findings correspond to those from Europe [13]. A comparable study in Wroclaw from 2017 to 2018 found ROSC in 21% of cases when a bystander performed CPR and 9% of cases when they did not [22].

This analysis revealed a statistically significant relationship between the initiation of CPR by a bystander before the arrival of the ambulance and the commencement of resuscitation efforts by the EMT upon arriving at the scene. The decision of the EMT supervisor to start resuscitation procedures is significantly influenced by whether the bystanders have performed CPR. Among the patients for whom CPR had been administered by a bystander, the EMT initiated resuscitation efforts in 67.89% of the cases, whereas among those where the bystander had not performed CPR, the EMT conducted resuscitation efforts significantly less often, in only 25.82% of the cases.

The decision to perform CPR by a bystander was influenced, among other factors, by the age of the victim. A statistically significant relationship was found between the age of the victim and the initiation of CPR by the bystander. The older the victim was, the less likely the bystanders were to perform CPR before the ambulance arrived.

In Bialystok, the total average time from the moment the dispatcher received the call to the arrival of the ambulance at the scene was 8 min and 30 s, with a median of 8 min (Q1 = 6 min, Q3 = 10 min). In Poland, the median time is similar, at 8 min in the Upper Silesia region and 7 min in Poznan [19,23]. The total time that best represents the final arrival time of the ambulance at the scene was likely influenced by several factors, including the correct execution of procedures by the dispatcher, the quality of contact with the person calling for the ambulance, the infrastructure of the ambulance station, the urgency displayed by the ambulance crew, the distance from the ambulance station to the incident location, and the traffic conditions.

No statistically significant difference was found between the time from the dispatcher’s receipt of the call to the arrival of the ambulance at the scene and the occurrence of ROSC in the patient. Studies conducted in other cities in Poland have not shown a statistically significant difference between the ambulance response time and the occurrence of ROSC in patients, either [16,22,24]. The average time from the receipt of the call to the arrival of the ambulance at the scene in the cases where ROSC occurred in Bialystok was 8 min and 27 s, while in the cases without ROSC, it was very similar at 8 min and 33 s. It is plausible to state that in other studies conducted in different cities in Poland, that time did not differ significantly, which is why no statistically significant differences have been recorded between the ambulance response time and the occurrence of ROSC in patients.

In Bialystok, during the initial ECG rhythm assessment performed by the EMT members, asystole was the most common first rhythm of cardiac arrest, followed by PEA, and VF or pVT. In Poland, studies conducted in Opole and Poznan showed similar frequencies of VF/pVT in the first rhythm assessment by the EMT members. The remaining rhythms consisted of asystole and PEA [23,25]. A higher percentage of VF or pVT was observed in Katowice [21]. Previous research has indicated that the earlier a heart rhythm is assessed in cases of OHCA, the more commonly a defibrillation is required. On average, the AED performed an early cardiac evaluation 3 min and 43 s faster than the EMT. In these cases, VF/VT was most frequently diagnosed [26].

Among the patients whose first observed rhythm during the ECG analysis was VF or pVT, ROSC was significantly more common as compared to the cases where the first rhythm was PEA or asystole. These findings are comparable with those obtained in Poland in 2018 [20]. Comparing the occurrence of ROSC among the patients with rhythms requiring defibrillation and other rhythms, a statistically significant relationship was also observed. When the first observed rhythm of cardiac arrest was VF or pVT, ROSC occurred in 58.62% of the cases. In contrast, among other cardiac arrest rhythms (asystole and PEA), ROSC occurred in only 26.56% of the cases. Those results align with the frequency of ROSC based on the first cardiac arrest rhythm observed in Katowice as well as in Europe as a whole [13,21].

For the group where the first rhythm of cardiac arrest was VF/pVT, the median age was 63 years. In the group where the first rhythm was asystole, the median age was significantly higher at 72 years. The highest median age was observed in the cases where the first rhythm was PEA, with a median age of 75 years.

Among the patients who experienced OHCA in VF/pVT rhythm, those who had undergone intubation had a statistically significantly lower rate of ROSC as compared to patients who had not undergone that procedure. In the cases of VF/pVT, defibrillation should be performed as a priority, and it should not be delayed [14]. When VF/pVT is present, the use of alternative airway management methods seems to be more advantageous than performing endotracheal intubation, as the latter procedure takes more time. Among the patients who experienced OHCA in asystole and PEA rhythms, endotracheal intubation did not significantly affect ROSC. In rhythms that do not require defibrillation, such as asystole and PEA, both endotracheal intubation and alternative airway management methods appear to have similar therapeutic significance. According to the guidelines of the European Resuscitation Council, endotracheal intubation should be performed by an experienced individual; in other cases, airway management can be achieved using a supraglottic airway device or a laryngeal mask [14]. So far, the analyses of the frequency of ROSC in relation to the performance of endotracheal intubation have primarily focused on all the groups of ECG rhythms without differentiating between those that require defibrillation and those that do not. These studies have shown that performing intubation in cases of OHCA does not significantly impact the occurrence of ROSC [27,28].

Among the individuals who experienced OHCA in Bialystok, ROSC was achieved in 36.41% of the cases during advanced life support (ALS) provided by the EMT, and the patients were subsequently transferred to hospital. A similar ROSC rate was observed throughout the Podlaskie Voivodeship and in the Katowice area [20,29]. Those results are significantly higher than the ROSC rate observed in Europe, which stands at 25.2% and has remained unchanged over the past few years [4,13].

This study has potential limitations. The analysis included dispatch records of patients who received medical assistance in Bialystok. This study did not cover rural areas, where access to EMTs is limited. In addition, this study did not cover other provincial cities in Poland, which may significantly limit the use of the presented results. Such studies should be conducted in all provincial cities in Poland, so that the results can be used to improve the functioning of the EMS. Another limitation of this study is the inaccurately completed medical documentation. Some cases had to be rejected from the analysis due to insufficient data contained in the rescue cards or their incorrect completion. Meticulous completion of the cards would allow for the analysis of a larger number of cases. Greater emphasis should be placed on training EMTs in the field of maintaining medical documentation, so that it is maintained in a conscientious and unified manner.

## 5. Conclusions

The most significant factors influencing the occurrence of ROSC in patients and the decision to initiate resuscitation efforts by the EMT are the initiation and execution of CPR by bystanders. It is essential to enhance public awareness and knowledge about performing CPR. Achieving that goal requires educating the community, providing first aid training, and conducting campaigns and informational activities. Furthermore, during the occurrence of rhythms such as VF/pVT that require defibrillation, the use of alternative methods for airway management, such as a mask or laryngeal tube, results in a higher occurrence of ROSC.

To better understand the factors that increase the occurrence of ROSC, continued scientific research on OHCA cases is essential, along with collaboration with international organisations by providing them with the compiled records of those cases.

## Figures and Tables

**Table 1:** Distribution of OHCA cases by gender and age.

Age	Women	Men	Total [%]
18–29 yo	2	12	1.81
30–39 yo	2	30	4.14
40–49 yo	13	44	7.37
50–59 yo	26	77	13.32
60–69 yo	42	110	19.79
70–79 yo	57	99	20.31
80–90 yo	112	91	26.39
Over 90 yo	32	17	6.34

yo—years old.

**Table 2:** CPR initiation by bystanders.

**The effect of CPR initiation by bystanders on the occurrence of ROSC and MEPs**				
	Frequency of CPR initiation by bystanders		ROSC ^1^ (*p* < 0.0001)	MEPs ^1^ (*p* < 0.0001)
	All patients (n = 787)	Patients without signs of death (n = 655)		
CPR	26.81% (n = 211)	29.01% (n = 190)	30.53% (58/190)	67.89% (129/190)
No CPR	36.34% (n = 286)	32.52% (n = 213)	2.35% (5/213)	25.82% (55/213)
No data	36.85% (n = 290)	38.47% (n = 252)	-	-
**The initiation of CPR by bystanders depending on the patient’s age (n = 487)**				
	CPR ^2^ (*p* < 0.0001)			
18–39 yo	58.82% (20/34)			
40–64 yo	53.06% (78/147)			
65–80 yo	43.14% (66/153)			
>80 yo	28.10% (43/153)			

CPR—cardiopulmonary resuscitation, MEPs—medical emergency procedures, ROSC—return of spontaneous circulation, yo—years old; ^1^ Pearson’s Chi-square test, ^2^ The Mann–Whitney test.

**Table 3:** Factors affecting the occurrence of ROSC.

**Cardiac Arrest Rhythms During the Initial Assessment ECG** **and the Occurrence of ROSC**			
	Frequency of cardiac arrest rhythms during the initial assessment ECG		ROSC ^1^ (*p* < 0.0001)
	All patients (n = 574)	Patients without signs of death and in which the EMT initiated MEPs (n = 314)	
Asystole	74.56% (428)	59.24% (n = 186)	24.73% (46/186)
PEA	15.33% (88)	22.29% (n = 70)	31.43% (22/70)
VF/pVT	10.10% (58)	18.47% (n = 58)	58.62% (34/58)
**The effect of intubation on the occurrence of ROSC** **in shockable and non-shockable rhythm of SCA**			
	Intubation		No intubation
Shockable rhythm of SCA (n = 81) ^2^ (*p* < 0.05)	58.73% (37/63)		83.33% (15/18)
Non-shockable rhythm of SCA (n = 311) ^3^ (*p* = 0.83)	28.57% (62/217)		29.79% (28/94)

ECG—electrocardiogram, PEA—pulseless electrical activity, pVT—pulseless ventricular tachycardia, ROSC—return of spontaneous circulation, VF—ventricular fibrillation; ^1^ Pearson’s Chi-square test, ^2^ Pearson’s Chi-square test, ^3^ Pearson’s Chi-square test.

## Data Availability

The data will be made available upon request to the corresponding author.

## Supplementary Materials

The following supporting information can be downloaded at: https://www.mdpi.com/article/10.3390/jcm13247765/s1, S1: Questionnaire on medical procedures of emergency medical teams.
