# Supplementary material for: Actions Taken by Bystanders During Sudden Cardiac Arrest: Analysis of Emergency Medical Service Documentation in Poland
Source: J Clin Med. 2024 Dec 19;13(24):7765. doi: 10.3390/jcm13247765 (PMC11677351; doi:10.3390/jcm13247765)
Supplement: Supplementary file 1 [file jcm-13-07765-s001.zip › jcm-3325232-supplementary.pdf]

Supplement S1. Questionnaire on medical procedures of emergency medical teams

1. Location of the Emergency Medical Team (EMT):
   - o Białystok, ul. Poleska 89
   - o Białystok, ul. Pogodna 22
   - o Białystok, ul. Wielkopolska 8
2. Address or description of the place of the incident:……………………………………..
3. Geographic coordinates of the place of the incident:……………………………………
4. Place of incident:
   - o Apartment / House
   - o Street
   - o Workplace
   - o Store
   - o Medical clinic
   - o Social welfare home
   - o Sobering-up room
   - o Other: ……..……………………………………………………………..………
5. Date:…………………………………………………………………………………….
6. Time of receipt of the call:……………………………………………………………….
7. Time of transfer of the call to the EMT:….……………………….
8. Departure time of the EMT:……………………………………….
9. Time of arrival at the place of call:…..…………………………………………………..
10. Team type:
    - o S – specialist
    - o P – basic
11. Urgency Code:
    - o 1 – first urgency code
    - o 2 – second urgency code
12. Person calling:
    - o Family member
    - o By-stander
    - o EMT
    - o Police

- Fire bridage
- Municipal Police
- Other: …………………………………………………………………………..

13. Reason for calling:
    - No breathing/suspected cardiac arrest
    - Unconscious, breathing preserved
    - Rattling breathing
    - Chest pain
    - Dyspnoea
    - Fainting
    - Traffic accident
    - Hitting a pedestrian
    - Other: …………………………………………………………………………..
14. Sex of the patient:
    - Man
    - Woman
15. Age of the patient: …………………………………………………………………..
16. Pregnancy (only woman)
    - Yes
    - No
17. Odor from the mouth:
    - Normal
    - Organic solvent
    - Alcohol
    - Inny: …………………………………………………………………………….
18. Injury:
    - Yes
    - No
19. ECG rhythm during examination of the injured person immediately after arrival at the place of the incident by the EMT:
    - Sinus rhythm
    - Supraventricular tachycardia
    - Ventricular tachycardia
    - Atrial fibrillation/flutter

- Atrioventricular block
- VF / VT
- Asystole
- PEA
- Pacemaker
- Acute coronary syndrome

20. ECG rhythm during examination of an injured person during performance of medical rescue procedures by the EMT:
    - Sinus rhythm
    - Supraventricular tachycardia
    - Ventricular tachycardia
    - Atrial fibrillation/flutter
    - Atrioventricular block
    - VF / VT
    - Asystole
    - PEA
    - Pacemaker
    - Acute coronary syndrome
21. Patient management, procedures performed by EMT members:
    - Suction
    - Bag valve ventilation
    - Oropharyngeal tube
    - Intubation
    - Respirator
    - Passive oxygen therapy
    - Defibrillation
    - External stimulation
    - Cardioversion
    - Heart massage
    - ECG
    - Teletransmission
    - Cervical collar
    - Orthopedic board
    - Vacuum mattress

- Immobilization
- Dressing
- Peripheral venous line
- Central venous line
- Catheterization
- Gastric tube
- Monitoring
- Mechanical chest compression device

22. In case of a cardiac arrest of the injured person before the arrival of the Emergency Medical Services, the performance of CPR elements by a by-stander:
    - Yes, chest compressions and rescue breaths
    - Yes, only chest compressions
    - Yes, only rescue breaths
    - No
    - No data
23. In case of a cardiac arrest of the injured person before the arrival of the EMT, the use of AED by a by-stander:
    - Yes
    - No
    - No data
24. Transfer of a patient in hospital:
    - Yes, the patient was given help, transferred to the hospital.
    - No, The patient died before being transferred to the hospital.
25. In case of death, it occurred:
    - Before EMT arrival
    - During medical procedures
26. Occurrence of signs of death
    - Yes
    - No
